# Editorial: Hypertension and Chronic Kidney Injury or Failure

**DOI:** 10.3389/fphys.2021.662737

**Published:** 2021-03-11

**Authors:** Oleg Palygin, Zhengrong Guan, Suttira Intapad, Jennifer C. Sullivan

**Affiliations:** ^1^Medical College of Wisconsin, Milwaukee, WI, United States; ^2^Division of Nephrology, Department of Medicine, University of Alabama at Birmingham, Birmingham, AL, United States; ^3^School of Medicine, Tulane University, New Orleans, LA, United States; ^4^Department of Physiology, Medical College of Georgia, Augusta University, Augusta, GA, United States

**Keywords:** hypertension, renal damage, chronic kidney diseases, therapeutic treatment, clinical studies

## Introduction

Chronic kidney disease (CKD) negatively impacts long-term health consequences by increasing the risk of cardiovascular pathologies and hastening the progression of kidney failure. As a result, CKD is overall recognized as a significant medical problem, and despite the improvements in renal based therapy to treat high blood pressure, glomerulonephritis, and diabetes, the burden and incidents of CKD are increasing worldwide (Haileamlak, 2018). The underlying disease processes responsible for the multiple pathological changes in the kidney are likely influenced by several different factors including, but not limited to, genetics, the side effects of prescription medication, social and environmental stressors, sex/gender, aging, and the severity of cardiorenal pathologies like hypertension. Thus, it is vital to unravel the mechanisms causing renal injury and subsequently develop more effective and targeted therapeutic interventions to prevent and hopefully delay or even reverse the progressive renal dysfunction. A Research Topic focused on hypertension and chronic kidney injury or failure is thus timely and essential, adding to a growing body of renal research, and providing a foundation for building more effective treatments for cardiorenal abnormalities.

## Reviews

The Research Topic contains two review articles that addressed hypertension-associated renal dysfunction. The first article provides an important overview on compensatory adaptations to a solitary functioning kidney (SFK), and outlines how these adaptations may contribute to renal damage and hypertension later in life (McArdle et al.). Among the main factors that determine greater risk for developing hypertension and kidney injury in SFK patients are alterations in the renal sympathetic nerves, the renal Renin-Angiotensin System (RAS), and the nitric oxide system. The authors suggest that these systems can potentially be targeted in SFK to reduce the progression of disease. The second review article explores the physiological and pathophysiological conditions manifested primarily by the α2-adrenoceptors activation on renal sodium transporters, vascular tone, and on immune cells in the context of hypertension (Hering et al.). The activation of adrenoceptors promotes interaction between the non-adrenergic cells in the kidney and immune system, critically modulating vascular tone and sodium balance. Further investigation is needed to clarify the pathophysiological role of α2-adrenoceptors in the complex mechanism of renal sympathetic nerve activity in hypertension. Together, these reviews provide valuable insights on the mechanisms responsible for the blood pressure regulation and the progression of CKD.

## Pharmacological Management of Chronic Kidney Disease

New strategies for an effective intervention to prevent or slow progressive kidney disease have the potential impact in clinical practice. A trio of original research studies utilized well-established rat models to evaluate new treatments to slow dyslipidemia- or hypertension-induced renal injury. The effects of gemfibrozil treatment, a lipid-regulating medication also known as fibrates, was examined on the progression of renal injury in the obese Dahl salt-sensitive (SS) leptin receptor mutant (SS^LepR^mutant) rat (Shields et al.). Both male and female rats develop dyslipidemia, progressive proteinuria, and glomerular injury. Treatment with gemfibrozil significantly reduced glomerular pathology and lipid accumulation, and improved renal function, indicating that reductions in cholesterol and triglyceride levels in the bloodstream may successfully inhibit the development of obesity-induced hypertension and renal damage. Next, the therapeutic potential for the novel selective phosphodiesterase 1 inhibitor (PDE_1_) BTTQ was tested in spontaneously hypertensive and Dahl SS rats (Dey et al.). PDE_1_ inhibition induced vasodilation that was accompanied by a significant reduction of blood pressure in both experimental models. The vasodilatory properties and reduction of peripheral vascular resistance proposed to underlie the benefits of PDE_1_ inhibitors could be effective for the treatment of arterial hypertension and CKD. Another article assessed alterations in glomerular density and renal fibrosis following the use of ivabradine, hyperpolarization-activated cyclic nucleotide-gated channel blocker, in experimental rat models of hypertension (Stanko et al.). Ivabradine attenuated kidney alterations induced by L-NAME treatment in hypertensive Wistar rats. Overall, these studies provide new knowledge regarding the therapeutic applications which may be used in the treatment of hypertension and CKD.

## Early Life Stress and Intrarenal Ras Imbalance

An original research article by Dalmasso et al. described the impact of maternal separation (MatSep), a chronic behavioral model that mimics the effects of early life stress, on the intrarenal RAS. The study suggests that male Wistar Kyoto rats exposed to MatSep display permanent changes in the renal microvascular architecture in response to intrarenal RAS imbalance. The authors conclude that chronic behavioral stress during childhood may promote changes in renal hemodynamics and will be critical determinant for the hormonal regulation of fluid and electrolyte balance and promote the development of renal diseases in adulthood.

## Clinical Studies

This Research Topic includes a clinical report focused on the use of a combination of unenhanced computed tomography (CT) and texture analysis (CTTA) for the assessment of differential renal function without radioisotope renography in patients with chronic unilateral obstructive upper urinary tract stones (Li et al.). The findings of this study revealed the advantages of the utilization of CTTA as a non-invasive method to identify and characterize renal parenchymal volume for the estimation of renal function. Overall, this method may help urologists determine optimal treatment strategies using unenhanced CT. The impact of the gut microbiome (GM) on hypertensive patients was the focus of another clinical report (Nagase et al.). Using cross-sectional data of the Shika population-based study (Karashima et al., 2018), authors found a correlation between specific gut bacteria composition and the prevalence of hypertension. Moreover, changing dietary habits to low salt in individuals with a particular GM was ineffective in preventing hypertension. Authors conclude that the treatment for gut dysbiosis and restoration of GM homeostasis might be essential to precede the rise in blood pressure and the development of hypertension.

## Conclusion

The Research Topic Hypertension and Chronic Kidney Injury or Failure highlight recent studies and summarize new insights on the mechanisms contributing to kidney injury progression. This collection of 8 articles includes basic and clinical research demonstrating the importance of understanding physiological function, the use of recently developed therapeutic agents, and implementing novel clinical approaches for the treatment of hypertension and chronic kidney diseases.

## Author Contributions

OP conceived the content and drafted the manuscript. JS, ZG, and SI revised and approved the final manuscript. All authors contributed to the article and approved the submitted version.

## Conflict of Interest

The authors declare that the research was conducted in the absence of any commercial or financial relationships that could be construed as a potential conflict of interest.
